# The Diagnostic-Measurement Method—Resting Energy Expenditure Assessment of Polish Children Practicing Football

**DOI:** 10.3390/diagnostics11020340

**Published:** 2021-02-18

**Authors:** Edyta Łuszczki, Anna Bartosiewicz, Katarzyna Dereń, Maciej Kuchciak, Łukasz Oleksy, Artur Stolarczyk, Artur Mazur

**Affiliations:** 1 Institute of Health Sciences, Medical College of Rzeszów University, 35-959 Rzeszów, Poland; abartosiewicz@ur.edu.pl (A.B.); kderen@ur.edu.pl (K.D.); 2 Institute of Physical Culture Sciences, Medical College of Rzeszów University, 35-959 Rzeszów, Poland; mkuchciak@ur.edu.pl; 3 Orthopaedic and Rehabilitation Department, Medical University of Warsaw, 02-091 Warszaw, Poland; loleksy@oleksy-fizjoterapia.pl (Ł.O.); artur.stolarczyk@wum.edu.pl (A.S.); 4 Institute of Medical Sciences, Medical College of Rzeszów University, 35-959 Rzeszów, Poland; drmazur@poczta.onet.pl

**Keywords:** children and adolescents, indirect calorimetry, metabolism, physical activity, resting energy expenditure

## Abstract

Establishing the amount of energy needed to cover the energy demand of children doing sport training and thus ensuring they achieve an even energy balance requires the resting energy expenditure (REE) to be estimated. One of the methods that measures REE is the indirect calorimetry method, which may be influenced by many factors, including body composition, gender, age, height or blood pressure. The aim of the study was to assess the correlation between the resting energy expenditure of children regularly playing football and selected factors that influence the REE in this group. The study was conducted among 219 children aged 9 to 17 using a calorimeter, a device used to assess body composition by the electrical bioimpedance method by means of segment analyzer and a blood pressure monitor. The results of REE obtained by indirect calorimetry were compared with the results calculated using the ready-to-use formula, the Harris Benedict formula. The results showed a significant correlation of girls’ resting energy expenditure with muscle mass and body height, while boys’ resting energy expenditure was correlated with muscle mass and body water content. The value of the REE was significantly higher (*p* ≤ 0.001) than the value of the basal metabolic rate calculated by means of Harris Benedict formula. The obtained results can be a worthwhile suggestion for specialists dealing with energy demand planning in children, especially among those who are physically active to achieve optimal sporting successes ensuring proper functioning of their body.

## 1. Introduction

In order to function properly, a human being requires a sufficient amount of energy, which is present is food, to be supplied. The human energy demand consists of resting energy expenditure (REE), exercise metabolism expenditures and body growth development energy expenditure. These three elements constitute total energy expenditure [1]. To determine the amount of energy needed to cover human energy demand and thus ensure that an even energy balance is achieved, the daily energy expenditure should be determined. For children, it is very important that the energy demand associated with the processes of growth and development of the young body cannot be omitted [2]. 

In addition, optimized energy intake in the diet is crucial for the population of young athletes. Energy expenditure associated with body development intensive physical activity also increases demand. Measurement or estimation of REE is usually the first step in determining energy demand, both in the general population and for people training in various sports disciplines [3]. Observations made by many authors confirm that the REE is higher in people with greater body weight, who are either overweight or obese [4,5]. Age is also a parameter that has been confirmed in numerous studies to have an influence on the REE [6]. In addition, the strong relationship between the REE and muscle mass has been the subject of research of many authors. The literature shows the FFM as the strongest indicator affecting the REE [7,8]. Adipose tissue is another component of the body composition and its effect on the REE is also widely analyzed. Some researchers attribute an important role to it in influencing energy expenditure [9], while other studies do not show such a relationship [10]. Furthermore, REE has been reported to be strongly associated with blood pressure (BP), independent of age and various anthropometric measures [11].

There are a very few reports in the literature regarding the assessment of REE using indirect calorimetry in a population of children, especially those who do sports, as well as the factors that may affect the obtained result. Researchers presented that the REE of the children increased with age, both in boys and girls and there were significant gender differences in the 12 to 17 age group [12]. In another study, researchers compared two groups of children and showed that the differences in the REEs of the studied children result from differences in body composition [13]. The issue of the impact of physical characteristics on the REE in young people is thoroughly described by Herrmann et al. who based their research on a meta-analysis carried out on many works. The authors make reference to previous studies, which show that the REE in children and adolescents is influenced by lean body mass, fat mass and its location, vascular fat and ethnic differences of the surveyed [14].

To the authors’ knowledge, no research has yet assessed the parameters that may affect resting energy expenditure in children doing football training in Poland. Therefore, the aim of the study was to assess the correlation between selected factors and the REE in this group. 

## 2. Materials and Methods 

### 2.1. Subjects

The research took place at the Laboratory for Innovative Research in Dietetics (Center for Innovative Research in Medical and Natural Sciences, University of Rzeszów, Rzeszów, Poland). The rooms had a controlled temperature between 22 to 25 °C. A case-study conducted during the 2018/2019 school year in Rzeszów, southeastern Poland involved healthy children aged 9 to 17 years old from a sports school (Sports Champions School). The time of the study was held between 4 February 2019 to 28 June 2019. In order to select the school, invitations were sent to the school principals of all (7 schools) of the sports schools in the area (Podkarpackie Voivodeship, Poland). All of the schools represented a similar student profile and offered a similar sports program. Out of those that agreed (3 schools), one school was selected using a randomized algorithm. Sample size was determined with the help of the EPI INFO (StatCalc) software. Assuming there were 2000 pupils in the sports schools in Podkarpackie Voivodeship, it was estimated that the sample should include 179 children, with a confidence level of 95% and 7% margin of error. This sample size was increased to minimize possible losses. The study was conducted in a randomly selected school. A multistage random cluster sampling method was used.

**Figure 1:**
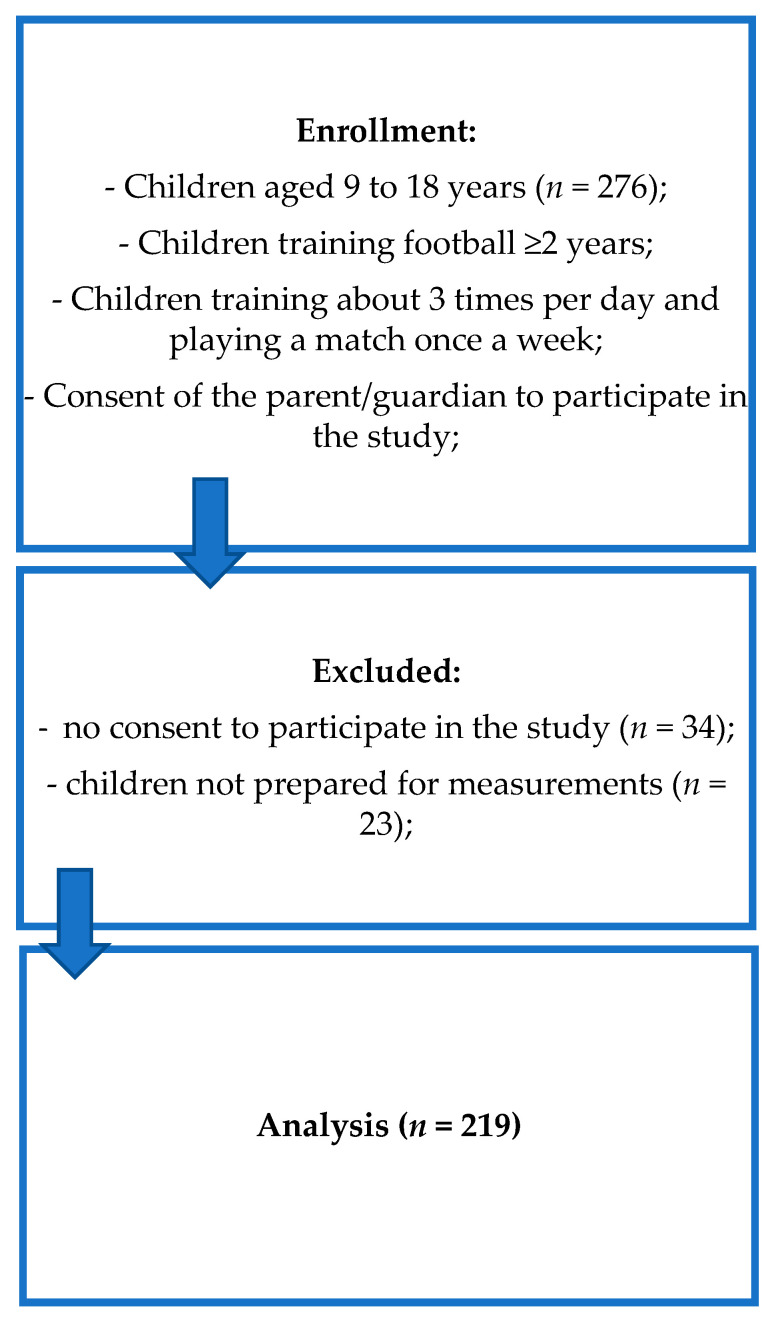
Selection of the study participants—own description.

Invitations to participate in the study were sent to 276 parents or guardians of children attending the selected school. The inclusion criteria were as follows: practicing football, training regularly, aged 9–18, consent of the parent/guardians to participate in the study. The selection of study participants is presented in Figure 1. All of the participants who gave permission were healthy, with a medical history showing no chronic diseases, no weight loss in the last 6 months and with no record of high temperature in the last month. In addition, they were not using any pharmacological or hormonal treatment. The study participants and their legal guardians received verbal and written information about the objectives, risks and benefits of the study. Finally, the study group consisted of 219 pupils (35 girls and 184 boys) aged 9 to 17.

### 2.2. Assessments

Prior to the measurement of indirect calorimetry, during an organizational meeting held with the pupils, the main purpose and the rules of the tests were explained. All recommendations concerning preparations for the study were outlined, including: having rest, refraining from the consumption of meals 12 hours before the test, refraining from drinking beverages with caffeine content for the last 48 hours before the test, as well as refraining from participation in a physical activity for the previous 12 hours. The method of conducting the study was explained in detail and each study participant had the opportunity to visit the test rooms beforehand and familiarize themselves with the equipment so that it did not raise concerns or cause anxiety in the researched group.

All of the measurements were carried out by experienced researchers. The participants gathered at the laboratory in the morning, between 6:00 and 9:00 a.m., were well-rested and refreshed, declaring compliance with all of the recommendations made to them during the organizational meeting.

#### 2.2.1. Resting Energy Expenditure Assessment by Indirect Calorimetry

Resting energy expenditure was measured by means of the indirect calorimetry method using a calorimeter (Fitmate MED, Cosmed, Rome, Italy). The device was calibrated according to the manufacturer’s instructions using a 3-liter syringe (Cosmed; P/N: C00600-01-11). In addition, each participant had the opportunity to acclimatize in the environment by lying flat for 30 min. REE was measured using a medical-grade cart (Cosmed, Rome, Italy) and silicone face masks (Cosmed, Rome, Italy). To assess REE, patients were asked to lay on their back on a couch (Norma II, Juventus, Poland) with a pillow placed under the head. Participants were instructed to lie still, avoid speaking and not fall asleep during the test. The Fitmate employs a turbine flowmeter for measuring ventilation and a galvanic fuel cell oxygen sensor for analyzing the fraction of oxygen in expired gases and uses standard metabolic formulas to calculate oxygen uptake the ratio of CO_2_ produced to O_2_ determines the respiratory quotient (RQ). The respiratory ratio (R) during the study was 0.85. The RQ value comes from averaging the values resulting from the oxidation of energy substrates, which are 1.0 for the protein, and 0.8 and 0.7 for the fat diet [15]. The respiratory ratio (R) during the study was 0.85. VCO_2_ (carbon dioxide volume) is not measured directly, it is estimated with a constant respiratory rate (RQ) of 0.85. Fitmate monitors oxygen uptake (VO_2_), fraction of O_2_ expired (FeO_2_), ventilation (Ve), heart rate (HR) and respiration rate (Rf). Estimation of REE (kcal/day) is possible thanks to the modified Weir equation: REE = [5.675 × VO_2_ + 1.593 × VCO_2_ − 21.7]; VO_2_ is the oxygen volume in the breath (mL/min) and VCO_2_ is the carbon dioxide output (mL/min) [16]. The Fitmate Med device was validated and showed very high reliability of the measurements obtained [17]. The results obtained using Fitmate Med are comparable to those obtained by the Douglas bag system, which uses a sensor to measure VCO_2_ [18]. A study by Campbell et al. examined the validity and reliability of the Fitmate device. On the first day, two 15-minute tests were performed, then on the second day (within a week after performing 1 test) another test was carried. To assess the reliability of the test, intraclass correlation coefficients (ICC) and standard error measurement (SEM) were used, while systematic error was analyzed by Anova. Relative consistency was accepted with the SEM and ICC values (0.981 and 0.946, responding during the day and in between). Moreover, no systematic error was found between measurements [17].

In order to properly use the device in the pediatric population, a request for guidance was sent to the manufacturer. According to the instructions, it was recommended to use disposable antibacterial filters with rubber mouthpieces to improve the mouth grip and limit the risk of air leaking by a reusable mask (a petite/pediatric size).

#### 2.2.2. Predictive REE Equation

In order to compare the results of resting energy expenditure obtained by means of the indirect calorimetry method with the results calculated using the ready-to-use formula, it was decided to calculate the REE using the common Harris Benedict formula, which makes use of data such as body weight, height and age [19]. In the case of healthy persons, the Harris Benedict formula, developed in 1919, is the most common. It is frequently considered as one of the most accurate formulas for basic energy demand calculations [19]. The equation was also based on a population of children and therefore can be used for studied group.

#### 2.2.3. Anthropometric Measurements, Body Composition and Body Mass Index

Prior to the indirect calorimetry study, all of the participants had anthropometric measurements and body composition analysis carried out. At the beginning, the participants were informed of the entire test procedure, including the need to empty the bladder if necessary, to minimize the risk of error during body composition analysis. The first stage of the study included measuring height using a height meter with an accuracy of 0.5 cm by means of Seca 213 portable stadiometer. The test participants were asked to remove their footwear and stand with their back to the stadiometer in an upright position during the measurement stage. The average of three measurements was used for the analysis.

Body weight and body composition were measured by the electrical bioimpedance method (6.25 kHz, 50 kHz, 90 µA) using a calibrated segment analyzer (Tanita MC-980 PLUS MA, Tokyo, Japan) with an accuracy of 0.1 kg/0.1%. Tanita MC 980 has approvals for medical use and meets the NAWI and CLASS III standards and the directive MDD 93/42/EEC, as well as the EU certificate CE0122 [20]. The results obtained using the Tanita Analyzer for studies involving children are consistent with those obtained from Dual Energy X-Ray Absorptiometry (DXA) [21,22,23,24]. The analyzer is equipped with 8 electrodes, 4 of which are built into the platform, while the others are in holders. The participants were asked to remove their footwear and socks, then they had the skin on their feet cleaned so that the measurement would be carried out correctly. All the test participants were in their underwear, stood still on the platform, in the designated places. In accordance with the Tanita MC-980 PLUS MA manual, the machine was set as vertically as possible to ensure accurate measurement. The device was set and adjusted so that the level indicator was in the center of the level meter. The participants stood on the platform barefoot, upright, with straight legs, placing their feet so that they touched the front and rear electrodes, making sure that the weight of the body was evenly distributed between both feet. In their hands, the examined person held handles positioned away from the body at an angle of 35°–40°. The purpose of the research was to estimate: body fat (%), lean body mass (kg), muscle mass (kg) and total body water (kg).

Body mass index (BMI) was calculated as body weight (kg)/height (m)^2^. Based on the BMI values, BMI percentiles were calculated for each participant. For analysis purposes, centile grids for BMI specific for age, sex and height developed under the project “Developing standards of blood pressure in children and adolescents in Poland, OLAF” were used [25]. On the basis of percentile grids, the examined children were characterized as: underweight (<5th percentile), normal body weight (between 5 and 85th centile), overweight (≥85 centile and <95 centile), obesity (≥95th centile).

Definitions of underweight, normal body weight, overweight and obesity were based on the Centre for Disease Control and Prevention recommendations [26].

#### 2.2.4. Arterial Blood Pressure

Blood pressure was measured three times according to the recommendations of the National High Blood Pressure Education Program Working Group in Children and Adolescents (NHBPEP) [27], by means of Welch Allyn 4200B-E2 blood pressure meter (Aston Abbotts, UK) with cuffs sized to fit the participants’ shoulder circumference. The average of three measurements was calculated for each person tested. Systolic and diastolic pressure values were used for the analysis.

### 2.3. Statistical Analysis

The study results were obtained using descriptive statistics: number (*n*), Me—median and standard deviation (SD). Both parametric and non-parametric tests were used to analyze the variables. The choice of the parametric test depended on fulfilling its basic assumptions, i.e., the conformity of the tested variable with normal distribution, which was verified by the Kolmogorov–Smirnov test. The student *t*-test (*t*) was used for normally distributed variables, alternatively a non-parametric Mann–Whitney U test was used. The correlation of the two variables was calculated with the spearman’s rho. Additionally, linear regression analysis was used. Using the stepwise forward regression procedure, the selection of factors in a statistically significant way describing the level of REE was made. The presented results were obtained using the principal component analysis (PCA) method of data reduction, which allowed the presentation of original observable variables using linear component combinations. The PCA was calculated by means of the main component method, with Oblim rotation, and the components were achieved by the Anderson–Rubin method. Statistical significance was established as a *p*-value less than 0.050. Calculations were performed with Statistica 10.0 tool (StatSoft, Inc., Tulsa, OK, USA).

### 2.4. Ethics

This research project was carried out in accordance with the Helsinki Declaration. The study was approved by the institutional Bioethics Committee at the University of Rzeszów (Resolution No. 2/01/2019, 2 January 2019) and all appropriate administrative bodies. Both the guardians and the participants gave their informed written consent to participate in the study.

## 3. Results

### 3.1. Characteristics of the Study Group

A total of 219 respondents aged 9 to 17 years were surveyed, 84% of whom were boys (*n* = 184) and 16% were girls (*n* = 35). The mean age of the respondents was 13.34 ± 2.24 years. The mean age of girls (14.46 ± 1.75 years) was significantly higher (*p* = 0.0016) than the mean age of boys (13.13 ± 2.27 years).

### 3.2. The Findings

The values obtained from the measurements taken are presented in Table 1.

Our study demonstrated that REE is significantly positively correlated with the majority of variables, both in girls and boys (Table 2). 

**Figure 2:**
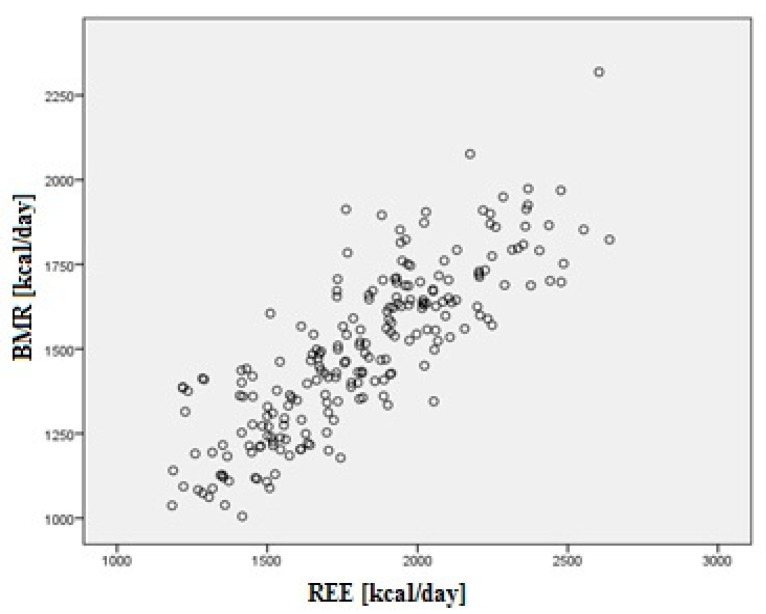
Correlation between the REE and the basal metabolic rate (BMR).

The REE is significantly positively correlated with the basal metabolic rate (BMR) calculated by means of Harris Benedict formula (*r* = 0.829; *p* < 0.0001). The value of the REE (mean 1808.03 ± 323.72 kcal) was significantly higher (*t* = 25.100; *p* < 0.0001) than the value of the BMR (average 1447.18 ± 239.34 kcal). Correlation between the REE and the BMR is presented in Figure 2.

We noticed that the REE differs significantly between girls (1616.71 kcal) and boys (1844.42 kcal). Therefore, linear regression was conducted separately for girls and boys and for all the test respondents.

Using factor analysis, (PCA—principal components analysis, Oblimin rotation—delta = 0), three main components were identified, explaining 90.7% of variance (the K-M-0 measure was 0.664). The first component was designated as the variable ‘body mass components’ and was created by body height (0.913), body weight (0.976), total body water (0.948) and muscle mass (0.967). The second component included the value of the fat tissue (0.914). The third component included systolic (0.520) and diastolic (0.774) pressure. New variables were generated by the Anderson–Rubin method and saved in *z*-score units.

The next step was the regression, which was performed using the stepwise forward regression procedure, first without taking into account the results of factor analysis (PCA) and then taking into account the new variables (body mass components, fat tissue and blood pressure) as well as the age and sex model.

The results of the stepwise forward regression indicated that the REE among all the respondents increased with muscle mass (β = 1.24) and was higher among boys (β = 0.14), decreased with age (β = −0.15) and decreased with increasing total body water (β = −0.31). In the group of girls, the REE was directly proportional to muscle mass and inversely proportional to body height. In boys, the REE, increased with muscle mass, but decreased with total body water.

The PCA method showed that among all the respondents the REE increased with variable body mass components (β = 0.81) and was higher in boys’ (β = 0.18). In girls, the REE increased with the increase in body mass components (β = 0.59), similarly in boys (β = 0.85). The result of general regression model for the selected parameters are presented in Table 3.

## 4. Discussion

To the best of authors’ knowledge, a very few test results are available in the literature regarding the effects of different factors on the resting energy expenditure in healthy children doing sport regularly. This study is one of a few undertaken in Poland using indirect calorimetry for calculating the REE in physically active children. There are only a few studies focusing on senior sports, or on people with chronic diseases [28], or obese [29]. The significant increase in interest in sport in Poland among children and adolescents requires researchers to present methods that allow to increase the level of training safety. One of these methods may be the correct assessment of energy expenditure and correlate it with the body components and blood pressure. This is a very important issue, as the optimization of energy intake in the diet is of fundamental importance in the population of young athletes, in whom, in addition to the caloric expenditure associated with the development of the body, the demand increases due to intensive physical activity. The value of the REE (mean 1808.03 ± 323.72 kcal) was significantly higher (*t* = 25.100; *p* < 0.0001) than the value of the BMR (average 1447.18 ± 239.34 kcal). For that reason, the measurement of REE is always recommended. 

The published results of research by foreign authors contain information which shows that the research covered mainly obese children [30,31] and children not participating in sports training processes [32]. Moreover, the content of the available thematic publications shows that the published results were based on a small number of respondents. The authors of this article based their findings on a much larger and broader in age range group of examined children and adolescents. Additionally, the results are a benchmark for the school involved in this study. The results are important for informing pupils and for comparison to other sports schools.

The study group consisted of 219 people, including 184 boys and 35 girls. This was due to the fact that the school selected for the study had a football profile, and it is still a more popular sport among men. The study showed significant differences in the REE levels between girls and boys in the study group. The boys had an average of 227.71 kcal higher REE than the girls. This is in line with the research available in literature [33,34].

The correlation between the REE and the selected parameters in body composition and between arterial pressure in most cases showed a positive link. With the increase of almost all parameters, the REE increased. The positive correlation concerned, the BMI for centile grids, among others. As BMI centiles increased, REE levels increased. This confirms the observations made by many authors that the REE is higher in people with greater body weight, who are also overweigh, or obese [4,35]. 

The results of the research also showed a positive correlation between the REE value and systolic and diastolic blood pressure (BP) in the study group. As the BP increased, the REE value increased. The most likely factor affecting this is blood catecholamine circulation [36]. A significant part of the literature indicates that people with higher BP have an increased level of catecholamines [37,38,39]. However, physical activity and training effects are inconsistent with the direct causal relationship between the REE and the BP. The REE tends to increase with aerobic fitness, while the BP and heart rate decrease [40,41].

Due to differences between the sexes, the linear regression model was conducted separately for girls and boys. The parameters that most influenced the REE value in girls were muscle mass (along with the increase in muscle mass in girls, the REE increased) and body height (the taller the girls, the lower the REE value). In the case of boys, it was also muscle mass (the more muscle mass, the higher the REE) and the total body water in the system (the higher the total body water, the lower the REE). The strong relationship between the REE and muscle mass has been the subject of research of many authors. The literature shows the FFM as the strongest indicator affecting the REE [7,8,42,43]. It has been shown that the FFM can have a strong impact on energy requirements. Webb in his study observed a strong correlation between the REE and the FFM in both men and women [44]. Cunnigham et al. found that FFM accounts for about 70% of the variation in the determination [45], and Lazzer et al. showed that FFM, as the metabolically active body component, represented 60% of the REE variability in the group of children and adolescents they examined [46]. However, research on this issue is still very ambiguous [47]. Adipose tissue is another component of the body composition and its effect on the REE is also widely analyzed. Some researchers attribute an important role to it in influencing energy expenditure [9,48,49], while other studies do not show such a relationship [10,50]. The results of this study did not confirm that adipose tissue has a significant effect on the REE. In the linear regression model, adipose tissue was not found as an independent factor affecting REE, which means that it has little effect, among other factors on the REE.

In the linear regression model, body height was also a strong predictor for girls—taller girls had lower REE. This is in line with research results showing that taller people have lower resting energy expenditure [51]. 

According to earlier studies in the pediatric population, higher REE in boys can be explained by a higher FFM compared to girls [52]. Sex remains a very strong factor affecting REE levels in children and adolescents. This can also be explained by higher proportions of skeletal glycolytic fibers [53], higher Na^+^-K^+^ ATPase activity [54] and other hormonal status [55]. 

There are also a number of potential limitations of the study that need to be taken into account when interpreting the results. Selection bias might have led to reduced generalizability as only one school was included in the study. In the future, the study should be expanded to include more schools from different geographical areas and other sports in addition to football. Furthermore, it would be worth paying attention to comparing the group of children who practice sport with children from elementary schools who do not exercise regularly. Another limitation of the study is the use of the electrical impedance method to assess body composition. Although it is used in literature on a large scale, the method of assessing body composition using densitometry would allow for even more precise results. In our study, a portable device (Fitmate Med) was employed. This may lead to errors when compared with the gold standard. Despite not measuring CO_2_ production, it is a very convenient in the clinical setting assuming a minimal error of analysis. 

## 5. Conclusions

The study enabled us to collect data on the factors that influenced REE in a group of children who train football regularly. Age, sex, body height and body mass components significantly influenced REE in this group. The obtained results can be a worthwhile suggestion for specialists dealing with energy demand planning in children, especially among those who are physically active to achieve optimal sporting successes ensuring proper functioning of their body.

## Figures and Tables

**Table 1:** Values of individual parameters in the study group.

		REE [kcal/day]	BMR Harris Benedict Formula [kcal/day]	Body Height [cm]	Body Weight [kg]	BMI Centiles	Fat Tissue [%]	Total Body Water [kg]	Muscle Mass [kg]	Systolic Pressure	Diastolic Pressure
Girls											
Average	1616.71		1417.02	163.66	54.05	50.63	25.36	29.53	38.28	111.91	67.89
SD	219.42		85.00	9.18	8.01	23.68	4.10	4.18	5.41	11.84	5.90
Me	1673.00		1419.43	164.00	54.00	45.00	25.20	29.20	37.90	110.00	69.00
Min	1219.00		1093.19	127.00	24.60	8.00	12.90	14.30	18.60	91.00	54.00
Max	1913.00		1577.71	181.00	69.80	90.00	34.50	37.50	48.60	141.00	83.00
Boys											
Average	1844.42		1512.43	162.91	52.37	49.47	17.58	31.84	40.96	113.09	63.63
SD	327.97		255.80	14.90	14.44	23.20	3.76	9.10	11.49	13.43	7.04
Me	1883.00		1552.16	166.00	53.85	49.00	16.90	32.75	42.10	113.00	63.00
Min	1183.00		1004.97	132.00	24.80	1.00	8.80	15.10	19.40	85.00	44.00
Max	2639.00		2318.72	191.00	102.20	96.00	34.00	58.80	75.70	158.00	83.00
Total											
Average	1808.03		1497.18	163.03	52.64	49.66	18.82	31.47	40.53	112.90	64.31
SD	323.72		239.34	14.12	13.61	23.22	4.76	8.54	10.79	13.17	7.04
Me	1807.00		1487.35	165.00	53.90	49.00	17.30	31.40	40.30	112.00	64.00
Min	1183.00		1004.97	127.00	24.60	1.00	8.80	14.30	18.60	85.00	44.00
Max	2639.00		2318.72	191.00	102.20	96.00	34.50	58.80	75.70	158.00	83.00
*p*	0.0001 *		0.0171 *	0.7888	0.5704	0.8866	0.0000 *	0.1358	0.1673	0.5323	0.0007 *

Me—median; SD—standard deviation; * indicates significant values (*p* < 0.05).

**Table 2:** Correlation between the resting energy expenditure (REE) and the selected factors.

Value		Girls	Boys
BMR Harris Benedict formula [kcal/day]	rho	0.541	0.843
	*p*	0.0008 *	0.0000 *
Age [years]	rho	0.130	0.707
	*p*	0.4574	0.0000 *
Body height [cm]	rho	0.304	0.815
	*p*	0.0757	0.0000 *
Body weight [kg]	rho	0.526	0.835
	*p*	0.0012 *	0.0000 *
BMI Centiles	rho	0.367	0.376
	*p*	0.0303 *	0.0000 *
Fat tissue [%]	rho	−0.026	−0.142
	*p*	0.8828	0.0539
Total body water [kg]	rho	0.636	0.822
	*p*	0.0000 *	0.0000 *
Muscle mass [kg]	rho	0.639	0.850
	*p*	0.0000 *	0.0000 *
Systolic pressure	rho	0.283	0.514
	*p*	0.0996	0.0000 *
Diastolic pressure	rho	0.434	0.233
	*p*	0.0092 *	0.0015 *

rho—Spearman’s Rho; *p*—significance of regression coefficient; * indicates significant values (*p* < 0.05).

**Table 3:** The result of general regression model for the selected parameters (independent variables were selected by forward stepwise regression procedure).

		Non-Standardized Coefficients		Standardized Coefficients	*t*	*p*	R^2^
		*B*	SE	β			
Total	(Constant)	732.91	116.78		6.28	0.0000 *	0.735
	Muscle mass [kg]	37.33	4.55	1.24	8.21	0.0000 *	
	Sex	125.82	35.68	0.14	3.53	0.0005 *	
	Age	−22.15	9.74	−0.15	−2.28	0.0239 *	
	Total body water [kg]	−11.88	5.33	−0.31	−2.23	0.0269 *	
Girls	(Constant)	2118.01	667.75		3.17	0.0033 *	0.478
	Muscle mass [kg]	45.16	10.01	1.11	4.51	0.0001 *	
	Body height [cm]	−13.63	5.90	−0.57	−2.31	0.0274 *	
Boys	(Constant)	851.56	46.42		18.35	0.0000 *	0.736
	Muscle mass [kg]	33.40	4.26	1.17	7.85	0.0000 *	
	Total body water [kg]	−11.78	5.37	−0.33	−2.19	0.0296 *	
Total (PCA)	(Constant)	1517.96	60.28		25.18	0.0000 *	0.716
	Body mass components	262.05	11.80	0.81	22.20	0.0000 *	
	Sex	157.63	32.13	0.18	4.91	0.0000 *	
Girls (PCA)	(Constant)	1671.41	33.04		50.59	0.0000 *	0.349
	Body mass components	243.45	57.87	0.59	4.21	0.0002 *	
Boys (PCA)	(Constant)	1833.18	12.74		143.89	0.0000 *	0.724
	Body mass components	262.92	12.02	0.85	21.87	0.0000 *	

SE—standard error; *B*—regression coefficient; *p*—significance of regression coefficient; β—standardized regression coefficient; *t*—Student *t* Test; * indicates significant values (*p* < 0.05); R^2^—R-squared values.

## Data Availability

The data presented in this study are available on reasonable request from the corresponding author: eluszczki@ur.edu.pl.

## Abbreviations

BMI	Body mass index
BMR	Basal metabolic rate
BP	Blood pressure
DXA	Dual Energy X-Ray Absorptiometry
FFM	Fat free mass
PCA	Principal components analysis
NHBPEP	National High Blood Pressure Education Program Working Group in Children and Adolescents
REE	Resting energy expenditure
RQ	Respiratory quotient
TEE	Total energy expenditure
WHO	World Health Organization
